# Quantifying Tolerance of a Nonlocal Multi-Qudit State to Any Local Noise

**DOI:** 10.3390/e20040217

**Published:** 2018-03-23

**Authors:** Elena R. Loubenets

**Affiliations:** Applied Mathematics Department, National Research University Higher School of Economics, Moscow 101000, Russia; elena.loubenets@hse.ru

**Keywords:** nonlocal N-qudit states, maximal Bell violation, tolerance to any local noise

## Abstract

We present a general approach for quantifying tolerance of a nonlocal *N*-partite state to any local noise under different classes of quantum correlation scenarios with arbitrary numbers of settings and outcomes at each site. This allows us to derive new precise bounds in *d* and *N* on noise tolerances for: (i) an arbitrary nonlocal *N*-qudit state; (ii) the *N*-qudit Greenberger–Horne–Zeilinger (GHZ) state; (iii) the *N*-qubit *W* state and the *N*-qubit Dicke states, and to analyse asymptotics of these precise bounds for large *N* and d.

## 1. Introduction

Nonlocality [1,2,3] of an *N*-qudit quantum state, *in the sense of its violation of a Bell inequality,* is a major resource for developing quantum information technologies. Conceptual and quantitative issues of Bell nonlocality in a general nonsignaling case have been analyzed in [4] and references therein. The main concepts and tools that were developed to describe and to study Bell nonlocality in a quantum case have been reviewed in [5]. (We further discuss only the notions of Bell nonlocality and locality and, therefore, mostly suppress the specification “Bell” before these terms.)

In quantum information applications, one, however, deals with noisy channels and, for a nonlocal *N*-qudit state $\rho_{d,N}$,d≥2,N≥2, it is important to evaluate amounts of noise not breaking the nonclassical character of its statistical correlations. Analytical and numerical bounds on the critical visibility of a nonlocal *N*-qudit state $\rho_{d,N}$ in a mixture *with white noise*:

(1) $$\left(1-\beta \right)\frac{I^{⊗N}}{d^{N}}+\beta \rho_{d,N},\;\;\;\beta \in \left[0,1\right],$$

have been intensively studied in the literature: (i) for a nonlocal two-qudit state—in [6,7,8,9,10] and references therein; (ii) for some specific quantum correlation scenarios and specific *N*-qubit states—in [11,12,13,14,15,16,17,18,19]; and (iii) for an arbitrary nonlocal *N*-qudit state $\rho_{d,N},$ N≥3,d≥3—in [20].

However, precise analytical bounds on the critical visibility of a nonlocal *N*-qudit state $\rho_{d,N}$ in a mixture

(2) $$\left(1-\beta \right)\zeta_{loc}+\beta \rho_{d,N},\;\;\;\beta \in \left[0,1\right],$$

with an arbitrary local noise (i.e., a noise described by a local *N*-qudit state $\zeta_{loc}$) and, more generally, bounds on the *tolerance* of a nonlocal *N*-qudit state $\rho_{d,N}$ to any local noise are not, to our knowledge, known in a general *N*-qudit case, though, for a nonlocal family of joint probabilities under a bipartite (N=2) correlation scenario, the similar concept—the resistance to noise—was introduced in [21] and further discussed in [5]. For the rigorous definition of the notion of the tolerance of a nonlocal state see Section 4.

We note that, for many quantum information applications based on Bell nonlocality, it is important to evaluate the maximal amount of noise tolerable by a nonlocal *N*-qudit state and this amount is determined specifically via the noise tolerance of a nonlocal state.

In the present paper, due to the general framework for Bell nonlocality developed in [4,22,23], we present a consistent approach to quantifying tolerance of a nonlocal *N*-partite quantum state to any local noise under different classes of quantum correlation scenarios with arbitrary numbers of settings and any spectral types of outcomes at each site. This allows us:

- to specify via parameters of an *N*-partite state the general analytical expressions for the noise tolerance of a nonlocal *N*-partite state (i) under $S_{1}\times \cdots \times S_{N}$-setting quantum correlation scenarios with any number of outcomes at each site and (ii) under all quantum correlation scenarios with arbitrary numbers of settings and outcomes per site;
- to derive new precise lower/upper bounds in *d* and *N* on the noise tolerances and the maximal amounts of tolerable local noise for: (i) an arbitrary nonlocal *N*-qudit state; (ii) the *N*-qudit Greenberger-Horne-Zeilinger (GHZ) state; (iii) the *N*-qubit *W* state and the *N*-qubit Dicke states and to analyse asymptotics of these precise new bounds for large *N* and d.

## 2. General N-Partite Bell Inequalities

Let us shortly recall the notion of a general multipartite Bell inequality [24] with arbitrary numbers of settings and outcomes per site. For the general framework on the probabilistic description of an arbitrary multipartite correlation scenario with any number of settings and any spectral type of outcomes at each site, see [25].

Consider a correlation scenario, where each *n*-th of *N* parties performs $S_{n}\geq 1$ measurements with outcomes $\lambda_{n}\in \left[-1,1\right]$ and every measurement at *n*-th site is specified by a positive integer $s_{n}=1,\ldots ,S_{n}$. For concreteness, we label an $S_{1}\times \cdots \times S_{N}$-setting scenario by $E_{S},$ where $S=S_{1}\times \cdots \times S_{N}.$

For a correlation scenario $E_{S}$, denote by $P_{\left(s_{1},\ldots ,s_{N}\right)}^{\left(E_{S}\right)}$ the joint probability distribution of outcomes $\left(\lambda_{1},\ldots ,\lambda_{N}\right)\in {\left[-1,1\right]}^{N}$ under an *N*-partite joint measurement induced by measurements $s_{1},\ldots ,s_{N}$ at the corresponding sites and by

(3) $$\begin{array}{ccc} B_{\Phi_{S}}^{\left(E_{S}\right)} & = & \sum_{s_{1},\ldots ,s_{{}_{N}}}{\langle f_{\left(s_{1},\ldots ,s_{N}\right)}\left(\lambda_{1},\ldots ,\lambda_{N}\right)\rangle }_{E_{S}},\\ \Phi_{S} & = & \{f_{\left(s_{1},\ldots ,s_{N}\right)}:{\left[-1,1\right]}^{N}\to R|s_{n}=1,\ldots ,S_{n},\;\;n=1,\ldots ,N\}, \end{array}$$

*a linear combination* of averages (expectations)

(4) $$\begin{array}{cc} & {\langle f_{\left(s_{1},\ldots ,s_{N}\right)}\left(\lambda_{1},\ldots ,\lambda_{N}\right)\rangle }_{E_{S}}\\ = & \int_{{\left[-1,1\right]}^{N}}f_{\left(s_{1},\ldots ,s_{N}\right)}\left(\lambda_{1},\ldots ,\lambda_{N}\right)P_{\left(s_{1},\ldots ,s_{N}\right)}^{\left(E_{S}\right)}\left(d\lambda_{1}\times \cdots \times d\lambda_{N}\right) \end{array}$$

of the most general form, specified for each *N*-partite joint measurement $\left(s_{1},\ldots ,s_{N}\right)$ by a bounded real-valued function $f_{\left(s_{1},\ldots ,s_{N}\right)}$ of outcomes $\left(\lambda_{1},\ldots ,\lambda_{N}\right)$∈${\left[-1,1\right]}^{N}$ at all *N* sites. Each linear combination (3) is specified by a family $\Phi_{S}=\left\{f_{\left(s_{1},\ldots ,s_{N}\right)}\right\}$ of these functions.

Depending on a choice of a function $f_{s_{1},\ldots ,s_{N}}$, an average (4) may refer either to the joint probability of events observed under this joint measurement at M≤N sites or to the expectation

(5) $$\langle \lambda_{1}^{\left(s_{1}\right)}\cdot \ldots \cdot \lambda_{n_{M}}^{\left(s_{n_{M}}\right)}\rangle_{E_{S}}=\int_{{\left[-1,1\right]}^{N}}\lambda_{1}\cdot \ldots \cdot \lambda_{n_{M}}P_{\left(s_{1},\ldots ,s_{N}\right)}^{\left(E_{S}\right)}\left(d\lambda_{1}\times \cdots \times d\lambda_{N}\right)$$

of the product of outcomes observed at M≤N sites or may have a more complicated form. In quantum information, the product expectation (5) is referred to as a correlation function.

Let the probabilistic description of a correlation scenario $E_{S}$ admit *a local hidden variable (LHV) model*, that is, all joint probability distributions $\left\{P_{\left(s_{1},\ldots ,s_{N}\right)}^{\left(E_{S}\right)},s_{n}=1,\ldots ,S_{n},n=1,\ldots ,N\right\}$ of this scenario admit the representation

(6) $$\begin{array}{cc} & P_{\left(s_{1},\ldots ,s_{N}\right)}^{\left(E_{S}\right)}\left(d\lambda_{1}\times \cdots \times d\lambda_{N}\right)\\ = & \int_{\Omega }P_{1,s_{1}}\left(d\lambda_{1}|\omega \right)\cdot \ldots \cdot P_{N,s_{{}_{N}}}\left(d\lambda_{N}|\omega \right)\nu_{E_{S}}\left(d\omega \right) \end{array}$$

in terms of a single probability distribution $\nu_{E_{S}}\left(d\omega \right)$ of some variables ω∈Ω and conditional probability distributions $P_{n,s_{n}}\left(\cdot |\omega \right),$ referred to as "local" in the sense that each $P_{n,s_{n}}\left(\cdot |\omega \right)$ at *n*-th site depends only on the corresponding measurement $s_{n}=1,\ldots ,S_{n}$ at this site. For the general statements on the LHV modelling, see Section 4 in [25].

In this case, each linear combination (3) of scenario averages satisfies the tight LHV constraints [24]:

(7) $$B_{\Phi_{S}}^{\inf}\leq B_{\Phi_{S}}^{\left(E_{S}\right)}{|}_{{}_{lhv}}\leq B_{\Phi_{S}}^{\sup}$$

with the LHV constants

(8) $$\begin{array}{ccc} B_{\Phi_{S}}^{\sup} & = & \underset{\lambda_{n}^{\left(s_{n}\right)}\in \left[-1,1\right],\forall s_{n},\forall n}{\sup}\;\sum_{s_{1},\ldots ,s_{{}_{N}}}f_{s_{1},\ldots ,s_{N}}\left(\lambda_{1}^{\left(s_{1}\right)},\ldots ,\lambda_{N}^{\left(s_{N}\right)}\right),\\ B_{\Phi_{S}}^{\inf} & = & \underset{\lambda_{n}^{\left(s_{n}\right)}\in \left[-1,1\right],\forall s_{n},\forall n}{\inf}\;\sum_{s_{1},\ldots ,s_{{}_{N}}}f_{s_{1},\ldots ,s_{N}}\left(\lambda_{1}^{\left(s_{1}\right)},\ldots ,\lambda_{N}^{\left(s_{N}\right)}\right). \end{array}$$

From relation (7), it follows that, in the LHV case,

(9) $$\left|\;B_{\Phi_{S}}^{\left(E_{S}\right)}{|}_{{}_{lhv}}\right|\leq B_{\Phi_{S}}^{lhv}=\max\left\{\left|B_{\Phi_{S}}^{\sup}\right|,\left|B_{\Phi_{S}}^{\inf}\right|\right\}.$$

Some of the LHV inequalities (7) may be fulfilled for a wider (than LHV) class of correlation scenarios. This is, for example, the case for the LHV constraints on joint probabilities following explicitly from nonsignaling of probability distributions. Moreover, some of the LHV inequalities (7) may be simply trivial, i.e., fulfilled for correlation scenarios of all types, not necessarily nonsignaling (For the latter general concept and its relation to the EPR (Einstein–Podolsky–Rosen) locality and Bell locality, see Sections 2 and 3 in [25].).

*Each of the tight LHV inequalities (7) that may be violated under a non-LHV scenario is referred to as a (general)* $S_{1}\times \cdots \times S_{N}$ *-setting Bell inequality.*

## 3. Quantum Violation

Let an $S_{1}\times \cdots \times S_{N}$-setting correlation scenario be performed on a quantum state ρ on a complex Hilbert space $H_{1}⊗\cdots ⊗H_{N}$. For this correlation scenario, every *N*-partite joint measurement $\left(s_{1},\ldots ,s_{N}\right)$ is described by the joint probability distribution

(10) $$\mathrm{tr}[\rho \left\{M_{1,s_{1}}\left(d\lambda_{1}\right)⊗\cdots ⊗M_{N,s_{N}}\left(d\lambda_{N}\right)\right\}],$$

where each $M_{n,s_{n}}\left(\cdot \right)$ is a normalized positive operator-valued (*POV*) measure, representing on $H_{n}$ a generalized quantum measurement $s_{n}$ at *n*-th site. For concreteness, we further specify this quantum correlation scenario by symbol $E_{\rho ,M_{S}}$, where $M_{S}=\left\{M_{n,s_{n}}\right\}$ is a collection of POV measures at all *N* sites.

Since the probabilistic description of a quantum correlation scenario does not need [1,2,3] to admit an LHV model, in a quantum case, Bell inequalities may be violated. The parameter [22]

(11) $$Y_{S_{1}\times \cdots \times S_{N}}^{\left(\rho \right)}=\underset{{}_{\Phi_{S},\;M_{S}}}{\sup}\frac{1}{B_{\Phi_{S}}^{lhv}}\left|B_{\Phi_{S}}^{\left(E_{\rho ,M_{S}}\right)}\right|\geq 1$$

specifies the maximal violation by an *N*-partite quantum state ρ of all general $S_{1}\times \cdots \times S_{N}$-setting Bell inequalities for any number of outcomes at each site and the parameter [22]

(12) $$Y_{\rho }=\underset{S_{1},\ldots ,S_{N}}{\sup}Y_{S_{1}\times \cdots \times S_{N}}^{\left(\rho \right)}\geq 1$$

specifies the maximal violation by an *N*-partite quantum state ρ of all general Bell inequalities for any numbers of settings and outcomes at each site.

From relations (3), (4), (10)–(12) it follows that, for any convex mixture $\rho =\sum \gamma_{i}\rho_{i},$ $\gamma_{i}\geq 0,$ $\sum_{i}\gamma_{i}=1,$

(13) $$\begin{array}{ccc} 1 & \leq & Y_{S_{1}\times \cdots \times S_{N}}^{\left(\rho \right)}\leq \sum_{i}\gamma_{i}Y_{S_{1}\times \cdots \times S_{N}}^{\left(\rho_{i}\right)}, \end{array}$$

(14) $$\begin{array}{ccc} 1 & \leq & Y_{\rho }\leq \sum_{i}\gamma_{i}Y_{\rho_{i}}. \end{array}$$

**Definition** **1.** *An N-partite quantum state ρ is referred to as $S_{1}\times \cdots \times S_{N}$-setting nonlocal [20] if it violates an $S_{1}\times \cdots \times S_{N}$-setting Bell inequality and overall nonlocal (or simply nonlocal) if it violates any of Bell inequalities.*

Clearly, an $S_{1}\times \cdots \times S_{N}$-setting nonlocal state is (overall) nonlocal but not vice versa. From Definition 1, relations (11), (12) and Proposition 6 in [22] it follows that an *N*-partite quantum state ρ is [20,22]:

- $S_{1}\times \cdots \times S_{N}$-setting nonlocal iff
  (15) $$Y_{S_{1}\times \cdots \times S_{N}}^{\left(\rho \right)}>1$$
  and $S_{1}\times \cdots \times S_{N}$-setting local iff
  (16) $$Y_{S_{1}\times \cdots \times S_{N}}^{\left(\rho \right)}=1.$$
- (overall) nonlocal iff
  (17) $$Y_{\rho }>1$$
  and fully Bell local [20] iff
  (18) $$Y_{\rho }=1.$$

For details and the one-to-one correspondence of relations (16) and (18) to the LHV modelling of the corresponding quantum correlation scenarios on an *N*-partite quantum state ρ, see Sections 5 and 6 in [22].

## 4. Tolerance to Any Local Noise

Let ρ be a nonlocal quantum state on $H_{1}⊗\cdots ⊗H_{N}$ and $S_{1},\ldots ,S_{N}$ be arbitrary numbers of measurement settings at the corresponding parties’ sites. Denote by

(19) $$\beta_{S_{1}\times \cdots \times S_{N}}^{\left(\rho \right)}\left(\zeta_{loc}\right)\in \left(0,1\right]$$

*the* $S_{1}\times \cdots \times S_{N}$*-setting critical visibility* of a nonlocal *N*-partite state ρ in a convex mixture with noise described by a local *N*-partite state $\zeta_{loc}:$

(20) $$\left(1-\beta \right)\zeta_{loc}\;+\beta \rho ,\;\;\;\beta \in \left[0,1\right].$$

In terminology specified in Section 3, the threshold $\beta_{S_{1}\times \cdots \times S_{N}}^{\left(\rho \right)}\left(\zeta_{loc}\right)$ means that a noisy state (20) is $S_{1}\times \cdots \times S_{N}$-setting nonlocal iff

(21) $$\beta \in (\beta_{S_{1}\times \cdots \times S_{N}}^{\left(\rho \right)}\left(\zeta_{loc}\right),1]$$

and $S_{1}\times \cdots \times S_{N}$-setting local iff

(22) $$\beta \in [0,\beta_{S_{1}\times \cdots \times S_{N}}^{\left(\rho \right)}\left(\zeta_{loc}\right)].$$

If $\beta_{S_{1}\times \cdots \times S_{N}}^{\left(\rho \right)}\left(\zeta_{loc}\right)=1,$ then a noisy state (20) is $S_{1}\times \cdots \times S_{N}$-setting local for all β∈[0,1]. For β=1, the latter implies that, though an *N*-partite state ρ is (overall) nonlocal, it does not violate any of $S_{1}\times \cdots \times S_{N}$-setting Bell inequalities, i.e., this state ρ is $S_{1}\times \cdots \times S_{N}$-setting local.

Let $L_{S_{1}\times \cdots \times S_{N}}^{\left(nonloc\right)}$ be the set of all $S_{1}\times \cdots \times S_{N}$-setting nonlocal *N*-partite states on $H_{1}⊗\cdots ⊗H_{N}$ and $L_{N}^{\left(nonloc\right)}⊃L_{S_{1}\times \cdots \times S_{N}}^{\left(nonloc\right)}$—the set of all (overall) nonlocal *N*-partite states.

**Definition** **2.** *For a nonlocal N-partite state $\rho \in L_{N}^{\left(nonloc\right)},$ we call*

(23) $$T_{S_{1}\times \cdots \times S_{N}}^{\left(\rho \right)}=\underset{\zeta_{loc}}{\sup}\beta_{S_{1}\times \cdots \times S_{N}}^{\left(\rho \right)}\left(\zeta_{loc}\right)\in \left(0,1\right]$$

*the $S_{1}\times \cdots \times S_{N}$-setting tolerance to any local noise. Otherwise expressed,*

(24) $$T_{S_{1}\times \cdots \times S_{N}}^{\left(\rho \right)}=\inf\left\{\beta \in \left[0,1\right]|\left(1-\beta \right)\zeta_{loc}\;+\beta \rho \in L_{S_{1}\times \cdots \times S_{N}}^{\left(nonloc\right)},\;\forall \zeta_{loc}\right\}.$$

Clearly, a noisy state (20) is $S_{1}\times \cdots \times S_{N}$-setting nonlocal for *any* local noise iff $\beta \in (T_{S_{1}\times \cdots \times S_{N}}^{\left(\rho \right)},1]$.

If $T_{S_{1}\times \cdots \times S_{N}}^{\left(\rho \right)}=1,$ then a nonlocal *N*-partite state $\rho \in L_{N}^{\left(nonloc\right)}$ is $S_{1}\times \cdots \times S_{N}$-setting local. Since, however, ρ is overall nonlocal, there exist numbers ${\tilde{S}}_{1},\ldots ,{\tilde{S}}_{N}$ of measurement settings at the corresponding sites for which this state is ${\tilde{S}}_{1},\ldots ,{\tilde{S}}_{N}$-setting nonlocal. For these settings, $T_{{\tilde{S}}_{1}\times \cdots \times {\tilde{S}}_{N}}^{\left(\rho \right)}<1.$

**Definition** **3.** *For a nonlocal N-partite state $\rho \in L_{N}^{\left(nonloc\right)},$ we call*

(25) $$T_{\rho }=\underset{S_{1},\ldots ,S_{N}}{\inf}T_{S_{1}\times \cdots \times S_{N}}^{\left(\rho \right)}\in \left(0,1\right)$$

*the overall tolerance (or simply tolerance) to any local noise.*

This definition implies that, for all $\beta \in (T_{\rho },1]$, a noisy state (20) specified for a nonlocal state $\rho \in L_{N}^{\left(nonloc\right)}$ is nonlocal for any local noise.

*The smaller is the value of the noise tolerance* $T_{\rho }$ *of a nonlocal N-partite state* ρ *, the greater is the maximal amount*

(26) $$M_{\rho }=1-T_{\rho }$$

*of a local noise of any type tolerable by this nonlocal state (in the sense that a noisy state (20) specified for a nonlocal state* ρ *is also nonlocal under all quantum correlation scenarios) and therefore, the greater is the robustness of nonlocality of a state* ρ *to any local noise.*

**Proposition** **1.** *Let ρ be a nonlocal N-partite state and $S_{1},\ldots ,S_{N}$ – arbitrary numbers of measurement settings at the corresponding sites. The $S_{1}\times \cdots \times S_{N}$-setting tolerance of a nonlocal state ρ to any local noise has the form*

(27) $$T_{S_{1}\times \cdots \times S_{N}}^{\left(\rho \right)}=\frac{2}{1+Y_{S_{1}\times \cdots \times S_{N}}^{\left(\rho \right)}}$$

*and the overall noise tolerance of a nonlocal state ρ is given by*

(28) $$T_{\rho }=\underset{S_{1},\ldots ,S_{N}}{\inf}T_{S_{1}\times \cdots \times S_{N}}^{\left(\rho \right)}=\frac{2}{1+Y_{\rho }},$$

*where $Y_{S_{1}\times \cdots \times S_{N}}^{\left(\rho \right)}$ is the maximal violation (11) by a nonlocal state ρ of all $S_{1}\times \cdots \times S_{N}$-setting general Bell inequalities and $Y_{\rho }$ – the maximal violation (12) by a nonlocal state ρ of all general Bell inequalities.*

**Proof.**  From relations (3), (4), and (11) and linearity in ρ of quantum probability distributions (10), it follows that

(29) $$\beta Y_{S_{1}\times \cdots \times S_{N}}^{\left(\rho \right)}\leq Y_{S_{1}\times \cdots \times S_{N}}^{\left(\left(1-\beta \right)\zeta_{loc}\;+\beta \rho \right)}+\left(1-\beta \right)Y_{S_{1}\times \cdots \times S_{N}}^{\left(\zeta_{loc}\right)}.\;$$

By Definition 2, for each $\beta \in [0,T_{S_{1}\times \cdots \times S_{N}}^{\left(\rho \right)}],$ there exists ${\tilde{\zeta }}_{loc}$ such that a noisy state (20) is $S_{1}\times \cdots \times S_{N}$-setting local. For this ${\tilde{\zeta }}_{loc},$ relation (16) implies

(30) $$Y_{S_{1}\times \cdots \times S_{N}}^{\left(\left(1-\beta \right){\tilde{\zeta }}_{loc}\;+\beta \rho \right)}=1.$$

In addition, $Y_{S_{1}\times \cdots \times S_{N}}^{\left({\tilde{\zeta }}_{loc}\right)}=1.$ Taking this into account in relations (29) and (30), we have

(31) $$\begin{array}{ccc} \beta Y_{S_{1}\times \cdots \times S_{N}}^{\left(\rho \right)} & \leq & Y_{S_{1}\times \cdots \times S_{N}}^{\left(\left(1-\beta \right){\tilde{\zeta }}_{loc}\;+\beta \rho \right)}+\left(1-\beta \right)Y_{S_{1}\times \cdots \times S_{N}}^{\left({\tilde{\zeta }}_{loc}\right)}=2-\beta \\ & \Leftrightarrow & \;\;\beta \leq \frac{2}{1+Y_{S_{1}\times \cdots \times S_{N}}^{\left(\rho \right)}} \end{array}$$

for each $\beta \in [0,T_{S_{1}\times \cdots \times S_{N}}^{\left(\rho \right)}]$. Therefore,

(32) $$T_{S_{1}\times \cdots \times S_{N}}^{\left(\rho \right)}\leq \frac{2}{1+Y_{S_{1}\times \cdots \times S_{N}}^{\left(\rho \right)}}.$$

On the other hand, for each $\beta >T_{S_{1}\times \cdots \times S_{N}}^{\left(\rho \right)},$ a noisy state (20) is $S_{1}\times \cdots \times S_{N}$-setting nonlocal for every $\zeta_{loc},$ so that by Equation (15) the relation

(33) $$Y_{S_{1}\times \cdots \times S_{N}}^{\left(\left(1-\beta \right)\zeta_{loc}\;+\beta \rho \right)}>1$$

holds for all $\zeta_{loc}$ and each $\beta >T_{S_{1}\times \cdots \times S_{N}}^{\left(\rho \right)}.$ In view of relation (11) and linearity in ρ of quantum probability distributions (10), this, in turn, implies that, for each $\zeta_{loc}$ and every $\beta >T_{S_{1}\times \cdots \times S_{N}}^{\left(\rho \right)},$ there exist (i) an $S_{1}\times \cdots \times S_{N}$-setting Bell inequality, specified in relation (7) by some family ${\tilde{\Phi }}_{S}=\Phi_{S}\left(\zeta_{loc},\beta \right)$ of functions in average (4) and (ii) quantum measurements, specified by some family ${\tilde{M}}_{S}=M_{S}\left(\zeta_{loc},\beta \right)$ of POV measures, such that

(34) $$\left|\left(1-\beta \right)B_{{\tilde{\Phi }}_{S}}^{\left(E_{\zeta_{loc},{\tilde{M}}_{S}}\right)}+\beta B_{{\tilde{\Phi }}_{S}}^{\left(E_{\rho ,{\tilde{M}}_{S}}\right)}\right|>B_{{\tilde{\Phi }}_{S}}^{lhv}$$

for all $\zeta_{loc}$ and all $\beta \in (T_{S_{1}\times \cdots \times S_{N}}^{\left(\rho_{d,N}\right)},1].$

Varying inequality (34) in $\zeta_{loc},$ implies that, for each *x*: $=B_{{\tilde{\Phi }}_{S}}^{\left(E_{\zeta_{loc},{\tilde{M}}_{S}}\right)}/B_{{\tilde{\Phi }}_{S}}^{lhv}\in \left[-1,1\right],$ there must exist *y*: $=B_{{\tilde{\Phi }}_{S}}^{\left(E_{\rho ,{\tilde{M}}_{S}}\right)}/B_{{\tilde{\Phi }}_{S}}^{lhv}\in$$\left[-Y_{S_{1}\times \cdots \times S_{N}}^{\left(\rho \right)},Y_{S_{1}\times \cdots \times S_{N}}^{\left(\rho \right)}\right]$ such that

(35) $$\left|\;(1-\beta )x+\beta y\right|>1$$

for all $\beta \in (T_{S_{1}\times \cdots \times S_{N}}^{\left(\rho \right)},1].$ However, this is possible if

(36) $$\frac{2-\beta }{\beta }<Y_{S_{1}\times \cdots \times S_{N}}^{\left(\rho \right)}\;\;\;\;\Leftrightarrow \;\;\;\;\beta >\frac{2}{1+Y_{S_{1}\times \cdots \times S_{N}}^{\left(\rho \right)}}.$$

Together with the condition that relation (35) holds for all $\beta >T_{S_{1}\times \cdots \times S_{N}}^{\left(\rho \right)}$ and definition (24) of the tolerance $T_{S_{1}\times \cdots \times S_{N}}^{\left(\rho \right)}$, Equation (36) implies

(37) $$T_{S_{1}\times \cdots \times S_{N}}^{\left(\rho \right)}\geq \frac{2}{1+Y_{S_{1}\times \cdots \times S_{N}}^{\left(\rho \right)}}.$$

Inequalities (32) and (37) prove relation (27). Expression (28) follows from Equations (27) and (12). ◻

For a bipartite correlation scenario with two settings per site, expression (27) for the $S_{1}\times \cdots \times S_{N}$-setting noise tolerance of a nonlocal *N*-qudit quantum state agrees with minimizing over all possible parties’ measurements on a two-qudit state of the resistance of a nonlocal family of scenario joint probability distributions to any local noise—the notion that was introduced in [21].

## 5. General Bounds

Let us evaluate due to relations (27) and (28) the noise tolerances for an arbitrary nonlocal state $\rho_{d,N}$ on ${\left(C^{d}\right)}^{⊗N}$.

In view of our results in [22,23,26], we have the following general precise upper bounds on the maximal violation $Y_{S\times \cdots \times S}^{\left(\rho_{d,N}\right)}$ of all S×⋯×S-setting Bell inequalities by an arbitrary nonlocal *N*-qudit state $\rho_{d,N},$d≥2, N≥2:

(38) $$\begin{array}{ccc} Y_{2\times \cdots \times 2}^{\left(\rho_{d,N}\right)} & \leq & \min\{d^{\frac{N-1}{2}},\;3^{N-1}\}, \end{array}$$

(39) $$\begin{array}{ccc} Y_{S\times \cdots \times S}^{\left(\rho_{d,N}\right)} & \leq & \min\{d^{\frac{S(N-1)}{2}},\;{\left(2\min\{d,S\}-1\right)}^{N-1}\},\;\;\;S\geq 3, \end{array}$$

under projective quantum measurements at all sites and

(40) $$Y_{S\times \cdots \times S}^{\left(\rho_{d,N}\right)}\leq {\left(2\min\{d,S\}-1\right)}^{N-1},\;\;\;S\geq 2,$$

under generalized quantum measurements at all sites.

The maximal violation $Y_{\rho_{d,N}}$ by a nonlocal *N*-qudit state $\rho_{d,N}$ of all general Bell inequalities satisfies the relation [26]

(41) $$Y_{\rho_{d,N}}\leq {\left(2d-1\right)}^{N-1},\;\;\;d\geq 2,N\geq 2$$

for either of the above types of quantum measurements.

Taking these upper bounds into account in expressions (27) and (28), we derive the following bounds on the S×⋯×S-setting noise tolerance $T_{S\times \cdots \times S}^{\left(\rho_{d,N}\right)}$ of an arbitrary nonlocal *N*-qudit state $\rho_{d,N},$ d≥2, N≥2:

(42) $$\begin{array}{ccc} T_{2\times \cdots \times 2}^{\left(\rho_{d,N}\right)} & \geq & \frac{2}{1+\min\{d^{\frac{N-1}{2}},\;3^{N-1}\}},\\ T_{S\times \cdots \times S}^{\left(\rho_{d,N}\right)} & \geq & \frac{2}{1+\min\{d^{\frac{S(N-1)}{2}},{\left(2\min\{d,S\}-1\right)}^{N-1}\}},\;\;S\geq 3, \end{array}$$

under projective quantum measurements at all sites and

(43) $$T_{S\times \cdots \times S}^{\left(\rho_{d,N}\right)}\geq \frac{2}{1+{\left(2\min\{d,S\}-1\right)}^{N-1}},\;\;\;S\geq 2,$$

under generalized quantum measurements at all sites.

The overall noise tolerance $T_{\rho_{d,N}}$ of an arbitrary nonlocal *N*-qudit state $\rho_{d,N}$ satisfies the relation

(44) $$T_{\rho_{d,N}}\geq \frac{2}{1+{\left(2d-1\right)}^{N-1}},\;\;\;\;d\geq 2,\;N\geq 2,$$

for either of above types of quantum measurements. For d→∞, this lower bound decreases to zero as $\frac{2}{{\left(2d\right)}^{N-1}}.$

From bound (43), it follows that, for an arbitrary S×⋯×S-setting nonlocal *N*-qudit state ${\tilde{\rho }}_{d,N}$, the maximal amount

(45) $$M_{S\times \cdots \times S}^{\left({\tilde{\rho }}_{d,N}\right)}=1-T_{S\times \cdots \times S}^{\left({\tilde{\rho }}_{d,N}\right)}$$

of tolerable local noise is upper bounded by

(46) $$M_{S\times \cdots \times S}^{\left({\tilde{\rho }}_{d,N}\right)}\leq \frac{{\left(2\min\{d,S\}-1\right)}^{N-1}-1}{{\left(2\min\{d,S\}-1\right)}^{N-1}+1},\;\;\;d\geq 2,\;N\geq 2,\;S\geq 2,$$

under all S×⋯×S-setting quantum correlation scenarios.

For d≤S, this upper bound does not depend on a number *S* of measurement settings per site:

(47) $$M_{S\times \cdots \times S}^{\left({\tilde{\rho }}_{d,N}\right)}\leq \frac{{\left(2d-1\right)}^{N-1}-1}{{\left(2d-1\right)}^{N-1}+1},\;\;\;N\geq 2,\;\;S\geq d\geq 2,$$

while, for d≥S, it does not depend on a qudit dimension d:

(48) $$M_{S\times \cdots \times S}^{\left({\tilde{\rho }}_{d,N}\right)}\leq \frac{{\left(2S-1\right)}^{N-1}-1}{{\left(2S-1\right)}^{N-1}+1},\;\;\;N\geq 2,\;\;d\geq S\geq 2.$$

For example, for two-qudit and three-qudit cases and two measurement settings per site, the general bound (48) implies the following upper bounds on the maximal tolerable noise:

(49) $$M_{2\times 2}^{\left({\tilde{\rho }}_{d,2}\right)}\leq \frac{1}{2},\;\;\;\;M_{2\times 2\times 2}^{\left({\tilde{\rho }}_{d,3}\right)}\leq \frac{4}{5},$$

for all dimensions d≥2.

## 6. N-Qudit GHZ State

Consider now bounds on the noise tolerances for the *N*-qudit Greenberger–Horne–Zeilinger (GHZ) state

(50) $$GHZ_{d,N}=\frac{1}{d}\sum_{j,j_{1}}{\left(|e_{j}\rangle \langle e_{j_{1}}|\right)}^{⊗N}$$

on ${\left(C^{d}\right)}^{⊗N}.$ Here, $\left\{e_{m},m=1,\ldots ,d\right\}$ is an orthonormal base in $C^{d}.$

For this *N*-qudit quantum state, the maximal Bell violation $Y_{S\times \cdots \times S}^{\left(GHZ_{d,N}\right)}$ of all S×⋯×S-setting Bell inequalities admit the upper bounds [22,23,26], which are more specific than the general bounds (38)–(41). Namely, for all d≥2,N≥2:

(51) $$\begin{array}{ccc} Y_{2\times \cdots \times 2}^{\left(GHZ_{d,N}\right)} & \leq & \min\{d^{\frac{N-1}{2}},\;3^{N-1},1+2^{N-1}\left(d-1\right)\},\\ Y_{S\times \cdots \times S}^{\left(GHZ_{d,N}\right)} & \leq & \min\{d^{\frac{S(N-1)}{2}},{\left(2S-1\right)}^{N-1},1+2^{N-1}\left(d-1\right)\},\;\;\;S\geq 3, \end{array}$$

under projective quantum measurements and

(52) $$Y_{S\times \cdots \times S}^{\left(GHZ_{d,N}\right)}\leq \min\{{\left(2S-1\right)}^{N-1},1+2^{N-1}\left(d-1\right)\},\;\;\;S\geq 2,$$

under generalized quantum measurements.

The maximal violation $Y_{GHZ_{d,N}}$ by the GHZ state of all general Bell inequalities satisfies the relation [22]:

(53) $$Y_{GHZ_{d,N}}\leq 1+2^{N-1}\left(d-1\right),\;\;\;d\geq 2,\;N\geq 2,$$

for either of the above types of quantum measurements.

In view of relations (51)–(53), for the *N*-qudit GHZ state, the general bounds (42)–(49) on the noise tolerances and the maximal amount of tolerable noise can be improved.

Taking relations (51)–(53) into account in expressions (27) and (28), we come to the following bounds for the S×⋯×S-setting noise tolerance of the *N*-qudit GHZ state for all d≥2,N≥2:

(54) $$\begin{array}{ccc} T_{2\times \cdots \times 2}^{\left(GHZ_{d,N}\right)} & \geq & \frac{2}{1+\min\{d^{\frac{N-1}{2}},3^{N-1},1+2^{N-1}\left(d-1\right)\}},\\ T_{S\times \cdots \times S}^{\left(GHZ_{d,N}\right)} & \geq & \frac{2}{1+\min\{d^{\frac{S(N-1)}{2}},{\left(2S-1\right)}^{N-1},1+2^{N-1}\left(d-1\right)\}},\;\;\;S\geq 3, \end{array}$$

under projective quantum measurements and

(55) $$T_{S\times \cdots \times S}^{\left(GHZ_{d,N}\right)}\geq \frac{2}{1+\min[{\left(2S-1\right)}^{N-1},1+2^{N-1}\left(d-1\right)]},\;\;\;S\geq 2,$$

under generalized quantum measurements.

The overall noise tolerance of the *N*-qudit state GHZ state satisfies the relation

(56) $$T_{GHZ_{d,N}}\geq \frac{1}{1+2^{N-2}\left(d-1\right)},\;\;d\geq 2,\;N\geq 2,\;$$

for either of the above types of quantum measurements.

From bound (54), it follows that, under 2×⋯×2-setting correlation scenarios with projective measurements at all sites, the maximal amount (45) of a local noise of any type tolerable by the *N*-qudit GHZ state is upper bounded by

(57) $$W_{2\times \cdots \times 2}^{\left(GHZ_{d,N}\right)}\leq \min\{\frac{d^{\frac{N-1}{2}}-1}{d^{\frac{N-1}{2}}+1},\;\;\frac{3^{N-1}-1}{3^{N-1}+1},\;\;\frac{2^{N-2}\left(d-1\right)}{2^{N-2}\left(d-1\right)+1}\}$$

for all d≥2,N≥2.

For example, for the three-qutrit GHZ state (N=3,d=3), the maximal amount of tolerable local noise $W_{2\times 2\times 2}^{\left(GHZ_{3,3}\right)}\leq \frac{2}{3}.$

By relation (56), under all quantum correlation scenarios, the maximal amount (26) of any local noise tolerable by the *N*-qudit GHZ state satisfies the bound

(58) $$W_{GHZ_{d,N}}\leq \frac{2^{N-2}\left(d-1\right)}{2^{N-2}\left(d-1\right)+1},\;\;\;d\geq 2,\;N\geq 2.$$

For the two-qudit GHZ state (N=2), this general bound gives

(59) $$W_{GHZ_{d,2}}\leq \frac{d-1}{d},\;\;\;d\geq 2.$$

### N-Qubit Case

Let us evaluate the noise tolerances for the *N*-qubit GHZ state.

As it has been proven in [23], under projective measurements at all sites, the maximal violation $Y_{2\times \cdots \times 2}^{\left(GHZ_{2,N}\right)}$ by the *N*-qibit state $GHZ_{2,N}$ of *all general*2×⋯×2-setting Bell inequalities coincides with the maximal violation $2^{\frac{N-1}{2}}$ by this state of all *correlation* 2×⋯×2-setting Bell inequalities and is, therefore, given by

(60) $$Y_{2\times \cdots \times 2}^{\left(GHZ_{2,N}\right)}{|}_{proj.meas}=2^{\frac{N-1}{2}}.$$

By relation (27), this implies that, under 2×⋯×2-setting quantum correlation scenarios *with projective quantum measurements* at all sites, the 2×⋯×2-setting noise tolerance of the *N*-qubit GHZ state is given by

(61) $$T_{2\times \cdots \times 2}^{\left(GHZ_{2,N}\right)}{|}_{proj.meas}=\frac{2}{1+2^{\frac{N-1}{2}}},\;\;\;N\geq 2,$$

while the maximal amount (57) of any local noise tolerable by the *N*-qubit GHZ state is

(62) $$W_{2\times \cdots \times 2}^{\left(GHZ_{2,N}\right)}{|}_{proj.meas}=\frac{2^{\frac{N-1}{2}}-1}{2^{\frac{N-1}{2}}+1},\;\;\;N\geq 2.$$

For N=2,3, this bound implies

(63) $$W_{2\times \cdots \times 2}^{\left(GHZ_{2,2}\right)}{|}_{proj.meas}=\frac{\sqrt{2}-1}{\sqrt{2}+1},\;\;\;\;\;W_{2\times \cdots \times 2}^{\left(GHZ_{2,3}\right)}{|}_{proj.meas}=\frac{1}{3}.$$

Furthermore, in view of Equations (12), (53) and (60), the maximal violation $Y_{GHZ_{2,N}}$ by the *N*-qubit GHZ state of *all general* Bell inequalities satisfies the relations

(64) $$2^{\frac{N-1}{2}}\leq Y_{GHZ_{2,N}}\leq 1+2^{N-1}$$

under all generalized parties’ quantum measurements.

Therefore, by relation (28), the overall noise tolerance $T_{GHZ_{2,N}}$ of the *N*-qubit GHZ state admits the bounds

(65) $$\frac{1}{1+2^{N-2}}\leq T_{GHZ_{2,N}}\leq \frac{2}{1+2^{\frac{N-1}{2}}},\;\;\;N\geq 2,$$

where the lower and upper bounds decrease with increasing N. This, in particular, implies that, under all quantum correlation scenarios, a mixture of the *N*-qubit state $GHZ_{2,N}$ *with any local noise* is nonlocal for all

(66) $$\beta >\frac{2}{1+2^{\frac{N-1}{2}}}\underset{N>>1}{≃}2^{-\frac{N-3}{2}}.$$

For comparison: a mixture of the *N*-qubit GHZ state *with white noise* is nonlocal [17] for all $\beta >2^{-\frac{N-1}{2}}.$

Due to inequalities (65), we derive the following bounds for the maximal amount $M_{GHZ_{2,N}}$ of a local noise of any type tolerable by the the *N*-qubit GHZ state:

(67) $$\frac{2^{\frac{N-1}{2}}-1}{2^{\frac{N-1}{2}}+1}\leq M_{GHZ_{2,N}}\leq \frac{2^{N-2}}{1+2^{N-2}},\;\;\;\;N\geq 2.$$

Hence, *with increasing of a number N of qubits, the robustness of nonlocality of the N-qubit GHZ state to any local noise increases.*

## 7. N-Qubit Dicke States

In this section, we evaluate the overall noise tolerance for the *N*-qubit Dicke states $|D_{N}^{\left(k\right)}\rangle \langle D_{N}^{\left(k\right)}|$ on ${\left(C^{2}\right)}^{⊗N}$ with k=1,…,N−1 excitations:

(68) |DN(k)〉=1Nk∑jπj(|0〉⊗(N−k)⊗|1〉⊗k).

Here, {|0〉,|1〉} is an orthonormal base in $C^{2},$ notation $\pi_{j}$ means a permutation in the tensor product of *k* vectors |1〉 and (N−k) vectors |0〉 and the binomial coefficient (Nk) gives the number of such permutations.

For example, for k=1, the Dicke state $|D_{N}^{\left(1\right)}\rangle$ constitutes the *N*-qubit *W* state

(69) $$\begin{array}{ccc} |W_{N}\rangle & = & \frac{1}{\sqrt{N}}(\underset{N}{\underset{︸}{|0\rangle ⊗\cdots ⊗|0\rangle ⊗|1\rangle }}+\underset{N}{\underset{︸}{|0\rangle ⊗\cdots ⊗|1\rangle ⊗|0\rangle }}\\ & & +\ldots +\underset{N}{\underset{︸}{|1\rangle ⊗\cdots ⊗|0\rangle ⊗|0\rangle }}). \end{array}$$

For N=3,k=2, the three-qubit Dicke state with two excitations has the form

(70) $$|D_{3}^{\left(2\right)}\rangle =\frac{1}{\sqrt{3}}(|0\rangle ⊗|1\rangle ⊗|1\rangle +|1\rangle ⊗|0\rangle ⊗|1\rangle +|1\rangle ⊗|1\rangle ⊗|0\rangle ).$$

Due to the general upper bound (41) and the value of violation by the Dicke state $|D_{N}^{\left(k\right)}\rangle$ of the specific Bell inequality introduced in [17], the maximal violation (12) by the Dicke state $|D_{N}^{\left(k\right)}\rangle$ of all general Bell inequalities satisfies the relation

(71) 1+2N−1(2−1)(Nk)≤YDN(k)≤3N−1,N≥2,k=1,…,N−1.

From relations (28) and (71), it follows that, for the Dicke state $|D_{N}^{\left(k\right)}\rangle ,$ the overall noise tolerance $T_{D_{N}^{\left(k\right)}}$ admits the bounds

(72) 21+3N−1≤TDN(k)≤11+2N−2(2−1)Nk

for all N≥2, k=1,2,…,N−1.

Therefore, under all quantum correlation scenarios, a mixture (20) of the *N*-qubit Dicke state $|D_{N}^{\left(k\right)}\rangle$ with any local noise is nonlocal for all

(73) β>11+2N−2(2−1)Nk.

For a large even N>>1 and $k=\frac{N}{2},$ the binomial coefficient (NN/2)≃N>>12N2πN, so that, by relation (73), a mixture of the Dicke state $|D_{N}^{\left(\frac{N}{2}\right)}\rangle$ with any local noise is nonlocal for all

(74) β>11+2N−2(2−1)NN/2≃N>>1422−11πN.

Specifying inequalities (72) for k=1, we have the following lower and upper bounds for the noise tolerance $T_{W_{N}}$ of the *N*-qubit *W* state (69):

(75) $$\frac{2}{1+3^{N-1}}\leq T_{W_{N}}\leq \frac{N}{N+2^{N-2}\left(\sqrt{2}-1\right)},\;\;\;N\geq 2.$$

Hence, a mixture (20) of the *N*-qubit *W* state with any local noise is nonlocal for all

(76) $$\beta >\frac{N}{N+2^{N-2}\left(\sqrt{2}-1\right)},\;\;\;\;N\geq 2.$$

## 8. Conclusions

In this article, we have presented a new general framework for quantifying noise tolerances of a nonlocal *N*-partite quantum state under different classes of quantum correlation scenarios with arbitrary numbers of settings and outcomes at each site.

This allowed us (i) to consistently specify two types (23) and (25) of noise tolerances of a nonlocal *N*-partite qudit state; (ii) to express them due to relations (27) and (28) in terms of the maximal violations (11) and (12) by this state of two classes of general Bell inequalities and (iii) to derive further the following new precise lower and upper bounds on the noise tolerances and the maximal amounts of tolerable local noise:

- bounds (42)–(49)—for an arbitrary nonlocal *N*-qudit state;
- bounds (54)–(59), (61), and (65)–(67)—for the *N*-qudit GHZ state, in particular, the *N*-qubit GHZ state;
- bounds (72)–(76)—for the *N*-qubit Dicke states and the *N*-qubit *W* state;

and to analyze their asymptotics for large *N* and *d*. We, in particular, prove that, with increasing of a number *N* of qubits, the robustness of nonlocality of the *N*-qubit GHZ state to any local noise increases.

To our knowledge, none of these analytical bounds has been reported in the literature. 
